# Supplementary material for: Hyperentanglement in Nanophotonic Systems With Discrete Rotational Symmetry
Source: Nanophotonics. 2026 Mar 20;15(6):e70048. doi: 10.1002/nap2.70048 (PMC13642552; doi:10.1002/nap2.70048)
Supplement: Supplementary file 1 [file NAP2-15-e70048-s001.pdf]

Supplementary Materials for

Hyperentanglement in Nanophotonic Systems with Discrete Rotational Symmetry

Lior Fridman[1,2], Amit Kam[2,3], Amir Sivan[1,2], Guy Sayer[1,2], Stav Lotan[1,2], and Guy Bartal[1,2]

[1]Andrew and Erna Viterbi Department of Electrical & Computer Engineering, Technion, Haifa 32000, Israel
[2]Helen Diller Quantum Center, Technion – Israel Institute of Technology, Haifa 32000, Israel
[3]Physics Department, Technion – Israel Institute of Technology, Haifa 32000, Israel

1 Nanophotonic modes in circular symmetry

When TM-polarized light couples into a sub-wavelength aperture, it excites a surface plasmon polariton (SPP) that propagates along the metal surface. In an asymptotic approximation, this excitation can be modeled as a superposition of point sources, each represented by a first-kind and first-order Hankel function, distributed along the aperture sides. Each point-source contribution propagates across the gold surface and decays exponentially in the direction normal to the surface. We further approximate each point-source far from the slit side as a plane wave in the form $E_z \propto e^{-k_z z - jk_{sp}r}$.

The point-spread function (PSF) of a slab nanophotonic system corresponds to a Bessel function of the first kind and zero order; hence, the eigenmodes of such a system take the form of cylindrical harmonics, expressed as $j_l(r)e^{jl\theta}$. It is advantageous to consider instead the finite Fourier transform of the PSF, which takes the form of a ring with a radius of k_{sp}. In this approach, the system modes are points on the ring with phase differences driven by the excitation itself.

When considering a circular excitation slit excited by circularly polarized light, the entire ring is excited, giving rise to excitation -

$$E_z \propto \frac{1}{2\pi}\int_0^{2\pi} e^{-k_z z - jk_{sp}(\cos\theta\, x + \sin\theta\, y)}\, d\theta = e^{-k_z z} J_0(k_{sp}r) \tag{1}$$

Using the relations between the in- and out-of-plane derived from Maxwell's equations in circular basis, we obtain the relation -

$$\begin{pmatrix} E_x + jE_y \\ E_x - jE_y \end{pmatrix} = \frac{k_z}{k_{sp}^2}\begin{pmatrix} \frac{dE_z}{dx} + j\frac{dE_z}{dy} \\ \frac{dE_z}{dx} - j\frac{dE_z}{dy} \end{pmatrix} = E_0\frac{\kappa}{k_{SP}}\begin{pmatrix} -J_{+1}(k_{sp}r) \\ J_{-1}(k_{sp}r) \end{pmatrix} = \begin{pmatrix} E_{\sigma^-} \\ E_{\sigma^+} \end{pmatrix} \tag{2}$$

We now consider an excitation with a phase front $\phi(x, y)$ where x,y are the in-plane coordinates with respect to the center of the excitation slit. In this case, each point source's relative phase is derived from the excitation itself and can be described as $E_z \propto e^{-k_z z - jk_{sp}r + j\phi(x,y)}$.

When considering an excitation with a phase front of $\phi = \theta \cdot l$ where $\theta = arctan(\frac{y}{x})$, namely an excitation carrying OAM which is quantified by a topological number l, when plugging such an excitation into Equation 1 and deriving Equation 2 we arrive at a general form for the electric field of the excitation in the circular symmetry -

$$\begin{pmatrix} E_z \\ E_{\sigma_-} \\ E_{\sigma_+} \end{pmatrix} \propto \begin{pmatrix} J_n(k_{sp}r) \\ -J_{n+1}(k_{sp}r) \\ J_{n-1}(k_{sp}r) \end{pmatrix} \tag{3}$$

Where n is the topological number that quantifies the TAM of the excitation, we note that the relations between the field components are deterministic, and all components share the same TAM.

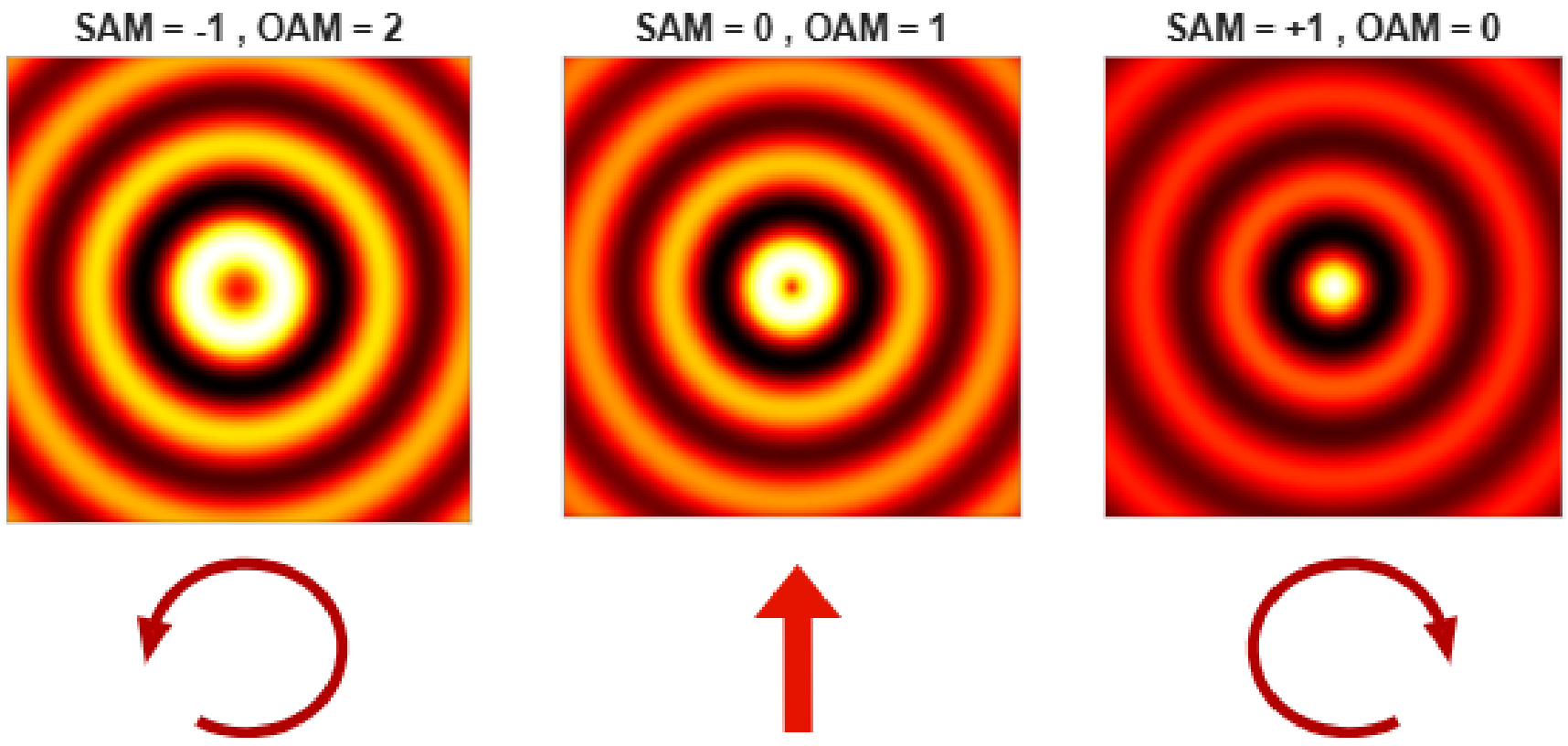

Figure S1: Field distribution of a nanophotonic mode - All field components share the same TAM but vary in its distribution into SAM and OAM. Each component has a defined SAM, thus making the OAM locked.

2 Total angular momentum in nanophotonics

The angular momentum of a photon can be decomposed into two distinct contributions: spin angular momentum (SAM) and orbital angular momentum (OAM). The SAM component is associated with the state of polarization, such that each photon carries an angular momentum of $\sigma = \pm 1\hbar$. The OAM, by contrast, originates from the azimuthal phase structure of the field. It is quantized by an integer topological charge l, defined for a helical phase front of the form $\phi = e^{jl\theta}$. This integer characterizes the number of 2π phase windings (or equivalently, the number of times the phase crosses zero) around the beam axis, with each photon in such a mode carrying $l\hbar$ of orbital angular momentum.

While considering a nanophotonic system, the SAM and OAM become strongly coupled such that the interplay between them is deterministically defined as seen in Equation 3. Although all the components carry the same TAM, they differ in their spatial profile since the out-of-plane field does not carry any SAM.

3 Angular momentum in discrete symmetry

We now consider a polygonal excitation slit, more specifically, a convex regular polygon. An excitation using such a coupler is equivalent to sampling of the circular PSF. It is more convenient to consider the Fourier transform, in which only points on the circle that correspond to the middle of the polygon sides are excited.

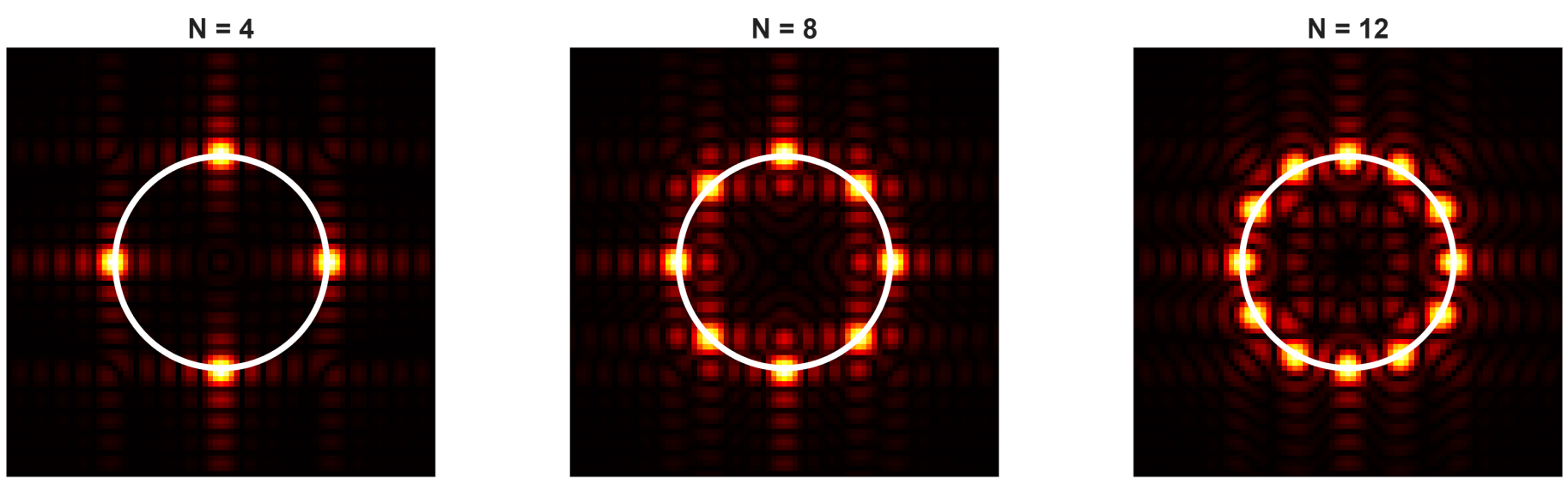

Figure S2: Excitation of regular polygonal couplers with varying N sides. A circle of radius k_{sp} is overlaid in white. Each point on the ring is treated as a point source propagating toward the polygon center, so the system's eigenmodes can be viewed as discrete samples of a circular slit.

As a result, the out-of-plane electric field can be approximated as a discrete sampling of Equation 1 and takes the form of -

$$E_z = e^{-|k_z|z} \sum_{n=1}^{N} A_n e^{-jk_{sp}[cos(\theta_n)x + sin(\theta_n)y]} \tag{4}$$

Where $\theta_n = \frac{2\pi}{N}n$ is the position of each side. The eigenmodes of such geometry are much more complex than those of a circular coupler and are highly dependent on the number of sides. Nevertheless, those modes can still be characterized by the angular momentum of the excitation itself since the spin-orbit locking, as shown in Equation 2, still holds.

We consider the SAM of such eigenmodes, and we find that it becomes ill-defined globally. For instance, we consider a square excitation slit, we find that the eigenmode out-of-plane electric field forms a lattice pattern in which every cell has a differing sign of SAM as was shown in [1]. More specifically, if we consider a circular polarization factor such as the polarization purity [2] -

$$\iint_{\text{Area}} \frac{\left|2\,\mathrm{Im}\left[E_x(x,y)\cdot E_y^*(x,y)\right]\right|}{\left|E_x(x,y)\right|^2+\left|E_y(x,y)\right|^2}\,dx\,dy \tag{5}$$

We find that its sign varies in each unit cell of the formed lattice, thus no global definition of the SAM of the mode can be made. In a similar manner, we refrain from defining the OAM of the eigenmode itself for the discrete case.

However, the complete vector field profile of each eigenmode is deterministically determined by the excitation's angular momentum, as demonstrated in [3], where our structured phase corresponds to a shift in the coupler sides. Analogous to Equation 3, each field component can be specified by a topological number derived from the TAM of the excitation. In this case, the eigenmode basis is limited to a dimension N, as shown in Equation 4, making the mapping non-injective and giving rise to aliasing-like behavior.

4 Discrete symmetry mode basis

Based on the discrete sampling approximation derived in Equation 4, we formally define the eigenmode basis for the N-sided polygonal geometry. Unlike the circularly symmetric case, where the eigenmode basis is infinite, the discrete rotational symmetry imposes a fundamental restriction, limiting the basis dimension to exactly N.

To fully define the basis, assume that the SPP excited from each side of the polygon has the same amplitude and that the size of the metal surface is sufficiently large, such that the spatial integration limits can be approximated as infinite.

Following our assumptions, we define the i-th eigenmode, denoted as E_i , by the following -

$$E_i(x,y) = \eta \sum_{n=1}^{N} e^{-jk_{sp}[\cos(\theta_n)x+\sin(\theta_n)y]}e^{j\theta_n\cdot i} \tag{6}$$

Here, we consider only the in-plane fields, thus omitting the dependence on z, and we omit the amplitude as well from the basis definition for clarity. The mode index i corresponds to its topological charge.

To demonstrate that these modes form an orthogonal basis, we calculate the spatial overlap integral between two modes with topological charges u and v over the in-plane dimensions -

$$\langle E_u|E_v\rangle = \iint_{-\infty}^{\infty} E_u^* E_v\,dx\,dy \tag{7}$$

Substituting the field expressions -

$$\begin{aligned}\langle E_u|E_v\rangle &= \eta^2 \sum_{n=1}^{N}\sum_{m=1}^{N} e^{j(\theta_n\cdot u-\theta_m\cdot v)} \iint_{-\infty}^{\infty} e^{-j(\mathbf{k}_n-\mathbf{k}_m)\cdot\mathbf{r}}\,dx\,dy \\ &= \eta^2 \sum_{n=1}^{N}\sum_{m=1}^{N} e^{j(\theta_n\cdot u-\theta_m\cdot v)} \int_{-\infty}^{\infty} e^{-j(\cos\theta_n-\cos\theta_m)k_{sp}x}\,dx \int_{-\infty}^{\infty} e^{-j(\sin\theta_n-\sin\theta_m)k_{sp}y}\,dy\end{aligned} \tag{8}$$

With our previous assumption, the spatial integrals lead to a delta function $\left(\frac{2\pi}{|k_{sp}|}\right)^2 \delta(\theta_n-\theta_m)$ taking the principal branch of $\theta_{n,m}\in[0,2\pi)$. This collapses the double sum into a single sum over the polygon sides. This result can be understood as a wave vector matching condition between the modes. Plugging this result yields -

$$\langle E_u|E_v\rangle = \eta^2\left(\frac{2\pi}{|k_{sp}|}\right)^2\sum_{n=1}^{N} e^{j\frac{2\pi}{N}n\cdot(u-v)} = \eta^2\left(\frac{2\pi}{|k_{sp}|}\right)^2 N\delta_{u,v} \tag{9}$$

Thus, we conclude that the eigenmode basis of each polygonal geometry is orthonormal with the normalization factor $\eta = \left[\left(\frac{2\pi}{|k_{sp}|}\right)\sqrt{N}\right]^{-1}$.

Finally, we observe the cyclical nature of the basis arising from the C_N discrete rotational symmetry of the polygon. The system is invariant under the discrete rotation group generated by angles $\Delta\theta = \frac{2\pi}{N}$. Mathematically, consider a mode with topological charge $l' = l + mN$ for any integer m, the phase factor imprinted at the n-th side, located at $\theta_n = \frac{2\pi}{N}n$, is:

$$e^{jl'\theta_n} = e^{j(l+mN)\frac{2\pi}{N}n} = e^{jl\theta_n}\cdot e^{j2\pi mn} \tag{10}$$

Since m and n are integers, the term $e^{j2\pi mn}$ equals unity. Consequently, the field distribution $E_z^{(l+mN)}$ is physically identical to $E_z^{(l)}$. This periodicity restricts the number of unique orthogonal modes to exactly N. This result provides the origin for the topological charge limitation discussed in the main text; it is a direct consequence of the representation theory of the C_N point group, which dictates that only N distinct irreducible representations exist.

5 Hyperentangled state derivation

We describe the generation of the hyperentangled state following the notations for the polarization states -

$$|H\rangle = \tfrac{1}{\sqrt{2}}\big(\,|\sigma_+\rangle + |\sigma_-\rangle\,\big)$$
$$|V\rangle = \tfrac{j}{\sqrt{2}}\big(-|\sigma_+\rangle + |\sigma_-\rangle\,\big)$$

Where $|H\rangle, |V\rangle$ are the linear polarization states, and $|\sigma_+\rangle, |\sigma_-\rangle$ are circular polarization states. From now on, we omit the normalization for the sake of brevity. We begin by modulating both photons with the same topological charge l to obtain -

$$|H\rangle|l\rangle \otimes |V\rangle|l\rangle = \big(\,|\sigma_+\rangle|l\rangle + |\sigma_-\rangle|l\rangle\,\big)\otimes\big(\,|\sigma_+\rangle|l\rangle - |\sigma_-\rangle|l\rangle\,\big)$$

In TAM -

$$(|l+1\rangle + |l-1\rangle)\otimes(|l+1\rangle - |l-1\rangle) = |l+1\rangle^{\otimes 2} - |l-1\rangle^{\otimes 2}$$

In plane -

$$\begin{aligned}\big(\,|\sigma_+\rangle|l\rangle - |\sigma_-\rangle|l+2\rangle\,\big)^{\otimes^2} - \big(\,|\sigma_+\rangle|l-2\rangle - |\sigma_-\rangle|l\rangle\,\big)^{\otimes^2} &=\\ |\sigma_+\rangle^{\otimes^2}|l\rangle^{\otimes^2} - |\sigma_+\rangle|l\rangle|\sigma_-\rangle|l+2\rangle - |\sigma_-\rangle|l+2\rangle|\sigma_+\rangle|l\rangle + |\sigma_-\rangle^{\otimes^2}|l+2\rangle^{\otimes^2}&\\ -|\sigma_+\rangle^{\otimes^2}|l-2\rangle^{\otimes^2} + |\sigma_+\rangle|l-2\rangle|\sigma_-\rangle|l\rangle + |\sigma_-\rangle|l\rangle|\sigma_+\rangle|l-2\rangle - |\sigma_-\rangle^{\otimes^2}|l\rangle^{\otimes^2}&\end{aligned}$$

We mark -

$$|A\rangle = |l\rangle \quad , \quad |B\rangle = |l-2\rangle \equiv |l+2\rangle$$

With this notation, we find -

$$\begin{aligned}&|\sigma_+\rangle^{\otimes^2}|A\rangle^{\otimes^2} - |\sigma_+\rangle|A\rangle|\sigma_-\rangle|B\rangle - |\sigma_-\rangle|B\rangle|\sigma_+\rangle|A\rangle + |\sigma_-\rangle^{\otimes^2}|B\rangle^{\otimes^2}\\ &-|\sigma_+\rangle^{\otimes^2}|B\rangle^{\otimes^2} + |\sigma_+\rangle|B\rangle|\sigma_-\rangle|A\rangle + |\sigma_-\rangle|A\rangle|\sigma_+\rangle|B\rangle - |\sigma_-\rangle^{\otimes^2}|A\rangle^{\otimes^2}\end{aligned}$$

Finally, we obtain -

$$\begin{aligned}&\left(\,|\sigma_+\rangle^{\otimes 2}-|\sigma_-\rangle^{\otimes 2}\,\right)\left(\,|A\rangle^{\otimes 2}-|B\rangle^{\otimes 2}\,\right)\\&-|\sigma_+\rangle\,|A\rangle\,|\sigma_-\rangle\,|B\rangle-|\sigma_-\rangle\,|B\rangle\,|\sigma_+\rangle\,|A\rangle+|\sigma_+\rangle\,|B\rangle\,|\sigma_-\rangle\,|A\rangle+|\sigma_-\rangle\,|A\rangle\,|\sigma_+\rangle\,|B\rangle\end{aligned} \tag{11}$$

6 Static rotation angle estimation

To estimate the static rotation angle, we consider two approximations. The first is the Huygens-Fresnel principle, in which the excitation gives rise to a first-order and first-kind Hankel function at every point along the coupler side. We assume that every Hankel is with the same amplitude and identical phase to the excitation. With such an assumption, each point along the coupler side contributes the following -

$$E_{point}=H_1^1\left(k_{sp}\sqrt{(x-x_s)^2+(y-y_s)^2}\right)e^{j\phi}$$

This is the Z component of the electric field of the point source, where ϕ is identical to the excitation phase. To calculate the excited field, we sum over all the coupler sides while considering the excited phase. All other field components are derived by Equation 2. In this approach, no analytical expression for the static rotation angle is achieved. The angle is calculated using the Hough transform and calculating the circular mean of angles detected.

Our second approach is the prism approximation, where we consider an excitation with a phase of $\phi = l\theta$. We approximate the Hankel sum as a single plane wave propagating from each edge toward the coupler center, and the imprinted phase by the excitation as a linear phase across the coupler edge, namely -

$$\phi = l\arctan\frac{y_s}{x_s}\approx l\frac{y_s}{x_s}$$

We now consider one such edge and approximate the excitation as -

$$E_{edge}\approx e^{jk_{sp}x}e^{-j\frac{2y}{L}l}$$

To find the rotation angle, we calculate the angle of the new plane of equal phase -

$$\begin{aligned}k_{sp}x-\frac{2ly}{L}&=0\\ \Rightarrow x&=\frac{2l}{k_{sp}L}y\end{aligned}$$

Thus, we find that the static rotation angle is $\theta_{stat}=arctan\left(\frac{2l}{K_{sp}L}\right)$. This calculation can be done on all edges without any loss of generality, yielding an approximation for the angle.

7 Experimental Feasibility and Characterization

To provide a complete assessment of the experimental realization, we present a comprehensive analysis of the experiment's feasibility. This includes a detailed noise model, a quantification of generation efficiency, and a rigorous description of the quantum characterization protocols for our hyperentangled state.

7.1 Noise Analysis and Decoherence Mechanisms

The experimental environment introduces several sources of noise that can affect the quality of the generated quantum states. We categorize these into coupling inefficiencies, photon tunneling, propagation losses, and fabrication imperfections.

7.1.1 Coupling Efficiency

Nanophotonic interfaces typically exhibit limited coupling efficiency between free-space photons and SPPs. Consequently, the quantum state inside the system is a mixed state dominated by vacuum terms. We model the polygonal in-coupler and circular out-coupler as probabilistic beam splitters.

Let κ_{in} and κ_{out} be the coupling efficiencies of the input and output couplers, respectively. The creation operator for a specific nanophotonic mode $a^\dagger_{mode}$ transforms as -

$$a^\dagger_{in} \to \kappa_{in} a^\dagger_{mode} + \sqrt{1-\kappa_{in}^2}\, a^\dagger_{loss} \tag{12}$$

Where $a^\dagger_{loss}$ represents photons that are reflected or scattered without coupling.

The target state for our scheme is the specific hyperentangled state $|\psi_{target}\rangle$ -

$$\begin{aligned} |\psi_{target}\rangle = &\left[|\sigma_+\rangle^{\otimes 2} - |\sigma_-\rangle^{\otimes 2}\right] \otimes \left[|A\rangle^{\otimes 2} - |B\rangle^{\otimes 2}\right] \\ &- |\sigma_+\rangle |A\rangle |\sigma_-\rangle |B\rangle - |\sigma_-\rangle |B\rangle |\sigma_+\rangle |A\rangle \\ &+ |\sigma_+\rangle |B\rangle |\sigma_-\rangle |A\rangle + |\sigma_-\rangle |A\rangle |\sigma_+\rangle |B\rangle \end{aligned} \tag{13}$$

Incorporating these coupling losses, the actual density matrix of the generated state can be expressed as a mixture of the desired hyperentangled component and a loss term -

$$\rho_{exp} = (\kappa_{in}\kappa_{out})^2 |\psi_{target}\rangle\langle\psi_{target}| + [1-(\kappa_{in}\kappa_{out})^2]\rho_{vac} \tag{14}$$

where ρ_{vac} encompasses the subspace of vacuum and single-photon states resulting from probabilistic coupling failure.

Due to these losses, the actual state reaching the detectors is highly mixed. However, our measurement scheme relies on coincidence counting, which effectively projects the state back onto the $|\psi_{target}\rangle$ subspace where two photons are successfully detected. While the probability of a successful two-photon coincidence scales with $(\kappa_{in}\kappa_{out})^2$, reducing the data acquisition rate, this process does not intrinsically degrade the entanglement fidelity of the detected pairs, provided the noise floor (dark counts and tunneling) is sufficiently low.

7.1.2 Direct transmission

A potential source of noise is photons passing through the gold film and reaching the detectors without being coupled to the nanophotonic modes. We calculate the transmission coefficient T_{tun} of the multilayer structure at normal incidence, which consists of four layers: Air (Layer 1), N-BK7 Glass substrate (Layer 2, $1\,mm$), Gold film (Layer 3, $220\,nm$), and Air (Layer 4).

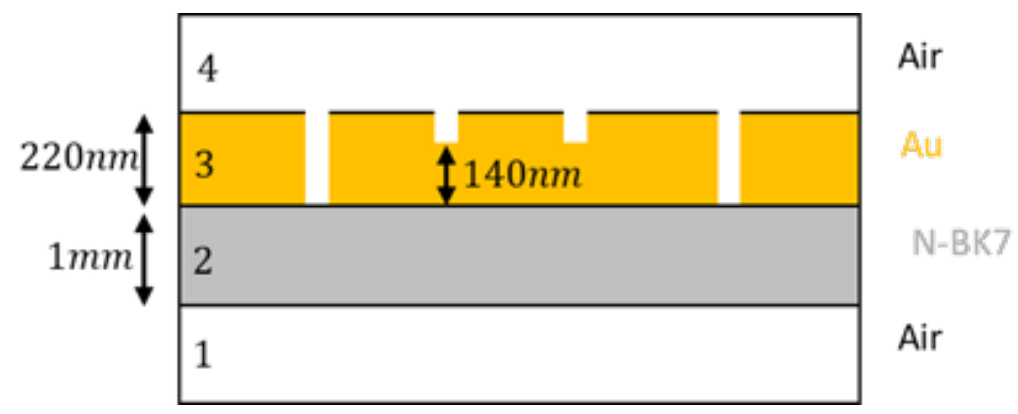

Figure S3: Cross-section of the multilayer nanophotonic platform used for the tunneling noise analysis. The structure consists of an Air superstrate, a $220\,nm$ Gold film, a $1\,mm$ N-BK7 glass substrate, and an Air substrate.

Using an effective transmission-line circuit method with the refractive indices $n_{NBK7} \approx 1.5106$ and $n_{Au} \approx 0.15598 + 4.9741i$ at $808\,nm$ yields -

$$|T_{tun}|^2 \approx 1.9 \cdot 10^{-5} \tag{15}$$

The likelihood of two photons tunneling through the platform is $|T_{tun}|^4 \approx 3.7 \cdot 10^{-10}$. In contrast, the coupling coefficient for one photon in and out of the plasmonic system is approximately $7 \cdot 10^{-3}$, resulting in an estimated probability of around $4.9 \cdot 10^{-5}$ for two photons to be measured. It is therefore highly improbable that the measured correlations stem from tunneling photons rather than coupled plasmons ($SNR \approx 10^7$).

7.1.3 Propagation Losses

SPPs propagating on a gold-air interface experience ohmic losses, leading to an exponential decay of the field as $e^{-\operatorname{Im}(k_{SPP})r}$. The dispersion relation is governed by $k_{SPP} = k_0\sqrt{\frac{\epsilon_{Au}}{1+\epsilon_{Au}}}$, where k_0 is the free-space wavevector. In our working regime, the permittivity of gold is $\epsilon_{Au} \approx -24.718 + 1.5517i$.

Crucially, since the generated state is confined to the in-plane dimensions, all constituent modes propagate with the identical propagation constant k_{SPP}, regardless of their specific spatial profile. Consequently, the propagation loss acts as a uniform global attenuation channel. Therefore, while ohmic losses reduce the overall brightness (efficiency), they do not introduce relative phase errors or decoherence between the superposed components of the state.

7.1.4 Fabrication Imperfections and Basis Deformation

The theoretical derivation of the discrete symmetry modes assumes equal coupling amplitude from all sides of the polygonal coupler. Ideally, for an N-sided polygon, the amplitude A_n from the n-th side is equal ($A_n = A_0$). However, fabrication imperfections such as variations in slit width or edge roughness can introduce random amplitude fluctuations, breaking the perfect C_N rotational symmetry.

We model this deformation by introducing a side-dependent perturbation δ_n such that the amplitude becomes $A_n = A_0(1+\delta_n)$, where δ_n is a random variable with zero mean and variance σ_{fab}^2. To relate the geometric variance of the slit width to the amplitude variance, we examine the transmission properties of subwavelength apertures. For a rectangular slit near the fundamental resonance, the transmittance T_{res} is dominated by the geometric aspect ratio. As described by Garcia-Vidal et al. [4], the resonant transmission can be approximated by:

$$T_{res} \approx \frac{3}{\pi}\frac{a_y}{a_x} \tag{16}$$

where a_x is the slit width and a_y is the length. This scaling implies that the relative change in transmission $\frac{\delta T}{T}$ is directly proportional to the relative change in slit width $\frac{\delta a_x}{a_x}$. Since the coupling amplitude A scales as $\sqrt{T}$, the relative amplitude variance is linearly proportional to the relative width variance to first order. This justifies our assumption that the variance of the amplitude scales linearly with the variance in fabrication dimensions, $\sigma_{fab} \approx \delta w/2w_0$.

The perturbed normalized eigenmodes $\left|\tilde{k}\right\rangle$ are superpositions of plane waves with these amplitudes. The overlap integral between two distinct modes k and m (where $k \neq m$) is given by -

$$\left\langle\tilde{k}\middle|\tilde{m}\right\rangle = \frac{1}{N}\sum_{n=1}^{N}|A_n|^2 e^{j\frac{2\pi}{N}(k-m)n} \tag{17}$$

Where we take $A_0 = 1$ for brevity. Substituting the perturbed amplitudes and using the approximation for small errors $(1+\delta_n)^2 \approx 1 + 2\delta_n$ -

$$\left\langle\tilde{k}\middle|\tilde{m}\right\rangle \approx \frac{1}{N}\sum_{n=1}^{N}(1+2\delta_n)e^{j\frac{2\pi}{N}(k-m)n} \tag{18}$$

Since $\sum_{n=1}^{N} e^{j\frac{2\pi}{N}(k-m)n} = 0$ for $k \neq m$, the zero-order term vanishes, leaving only the stochastic term -

$$\left\langle\tilde{k}\middle|\tilde{m}\right\rangle \approx \frac{2}{N}\sum_{n=1}^{N}\delta_n e^{j\frac{2\pi}{N}(k-m)n} \tag{19}$$

To estimate the magnitude of this cross-talk, we calculate the expected squared modulus of the overlap, which is the variance of the sum -

$$E\left[|\left\langle\tilde{k}\middle|\tilde{m}\right\rangle|^2\right] = E\left[\frac{4}{N^2}\sum_{n=1}^{N}\sum_{r=1}^{N}\delta_n\delta_r e^{j\frac{2\pi}{N}(n-r)(k-m)}\right] = \frac{4}{N}\sum_{n=1}^{N}E[\delta_n^2] = \frac{4}{N}\sigma_{fab}^2 \tag{20}$$

For a typical modern focused ion beam (FIB) fabrication system, the nominal beam resolution is approximately $4.0\,nm$. Accounting for both resolution limits and ion scattering (roughness $\sim 5\,nm$), the effective edge fluctuation is roughly $6.4\,nm$ [5]. Given a slit width of $\approx 200\,nm$, the relative amplitude fluctuation is $\sigma_{fab} \approx 0.016$. For a square slit ($N = 4$), this yields an expected cross-talk error of roughly 10^{-4}.

7.1.5 Phase Noise

The generation of high-fidelity hyperentangled states requires the preservation of a rigid phase relationship between the N constituent plasmonic modes. We model the total electric field at the geometric center as a superposition of contributions from each slit n as $E_{total} = \sum_{n=1}^{N} A_n e^{i(\Phi_0 + \delta\phi_n)}$, where Φ_0 is the ideal phase accumulation and $\delta\phi_n$ represents the deviation due to system imperfections. We analyze the three dominant stochastic contributions to $\delta\phi_n$ -

- **Structural Fabrication Variance -** Fabrication errors in the FIB process introduce a static path length difference. Let $\vec{r}_n$ be the ideal position vector of the n-th slit and $\delta\vec{r}_n$ be the stochastic displacement vector, assumed to be normally distributed with variance σ_{pos}^2. The resulting phase shift is $\delta\phi_{fab,n} = \vec{k}_{SPP} \cdot \delta\vec{r}_n$. For a radial displacement $\delta r_n \sim \mathcal{N}(0, \sigma_{pos}^2)$, the structural phase variance is:

$$\sigma_{fab}^2 = \left(\frac{2\pi}{\lambda_{SPP}} \sigma_{pos} \right)^2 \tag{21}$$

  Given $\lambda_{SPP} \approx 790\,nm$ and a typical FIB write precision of $\sigma_{pos} = 5\,nm$, we obtain $\sigma_{fab} = 0.040$ rad.

- **Excitation Alignment Gradient -** Ideally, the pump beam is incident normal to the surface ($\theta_{tilt} = 0$). A misalignment angle θ_{tilt} introduces a geometric optical path difference. We define the in-plane component of the incident wave vector as $\vec{k}_{\parallel} = k_0 \sin(\theta_{tilt})(\cos\beta_{tilt}\hat{x} + \sin\beta_{tilt}\hat{y})$ and the position of the n-th slit as $\vec{r}_n = R(\cos\alpha_n\hat{x} + \sin\alpha_n\hat{y})$. The phase deviation is the scalar product $\delta\phi_n = \vec{k}_{\parallel} \cdot \vec{r}_n$ -

$$\begin{aligned} \delta\phi_{align,n} &= k_0 R \sin(\theta_{tilt})[\cos\alpha_n \cos\beta_{tilt} + \sin\alpha_n \sin\beta_{tilt}] \\ &= k_0 R \sin(\theta_{tilt}) \cos(\alpha_n - \beta_{tilt}) \end{aligned} \tag{22}$$

  The maximum deviation is independent of the azimuthal angle β_{tilt} and is achieved for opposite sides of the structure (separated by $2R$), yielding a maximum optical path difference of $\Delta L_{opt} \approx 2R\sin(\theta_{tilt})$. Assuming a high-precision optical mount with residual tilt $\theta_{tilt} \approx 0.1°$ (1.7 mrad) and a structure radius $R = 15\,\mu m$, this path difference results in a maximum phase spread of $2k_0 R \sin(\theta_{tilt}) \approx 0.408$ rad. The corresponding standard deviation of this sinusoidal distribution is $\sigma_{align} \approx 0.144$ rad.

In a realistic experimental setting, these noise sources are statistically independent and uncorrelated. Therefore, the total phase uncertainty is best estimated by the quadrature sum of the individual standard deviations, and the aggregate phase noise is -

$$\sigma_{total} = \sqrt{\sigma_{fab}^2 + \sigma_{align}^2} \approx \sqrt{0.040^2 + 0.144^2} \approx 0.149 \text{ rad} \tag{23}$$

This corresponds to a phase uncertainty of approximately 8.6°.

8 Simulation of Noise Channels and Fidelity Estimation

To quantitatively validate the impact of the noise sources discussed previously, we performed a numerical Monte Carlo simulation.

Methodology

We simulate the full complex electric field distribution for the spatial modes. Each mode is generated as a superposition of N plane waves originating from the polygon sides, propagating towards the center, as defined by the discrete symmetry derivation Equation 6.

The simulation procedure is as follows -

- **State Construction -** The hyperentangled state vector is constructed in the full Hilbert space defined by the polarization ($\sigma_\pm$) and the continuous spatial coordinates (x, y). We focused specifically on the hyperentangled term -

$$|\psi\rangle \propto (|RR\rangle - |LL\rangle) \otimes (|AA\rangle - |BB\rangle) \tag{24}$$

- **Noise Injection -** Stochastic errors are introduced to the N plane wave components.

– **Amplitude Noise -** $A_n \to A_n(1+\delta_A)$, where $\delta_A \sim \mathcal{N}(0, \sigma_{amp}^2)$.
– **Phase Noise -** $\phi_n \to \phi_n + \delta_\phi$, where $\delta_\phi \sim \mathcal{N}(0, \sigma_\phi^2)$.

- **Spatial Fidelity Calculation -** The fidelity $F = |\langle \psi_{ideal} | \psi_{noisy} \rangle|^2$ is computed by numerically integrating the spatial overlap of the generated fields over a grid spanning $\pm 10\lambda_{spp}$ around the structure's center.

Results

We mapped the fidelity landscape over a parameter space covering phase noise up to 0.5 rad and amplitude noise up to 0.5 (relative std). The results are visualized in Figure S4.

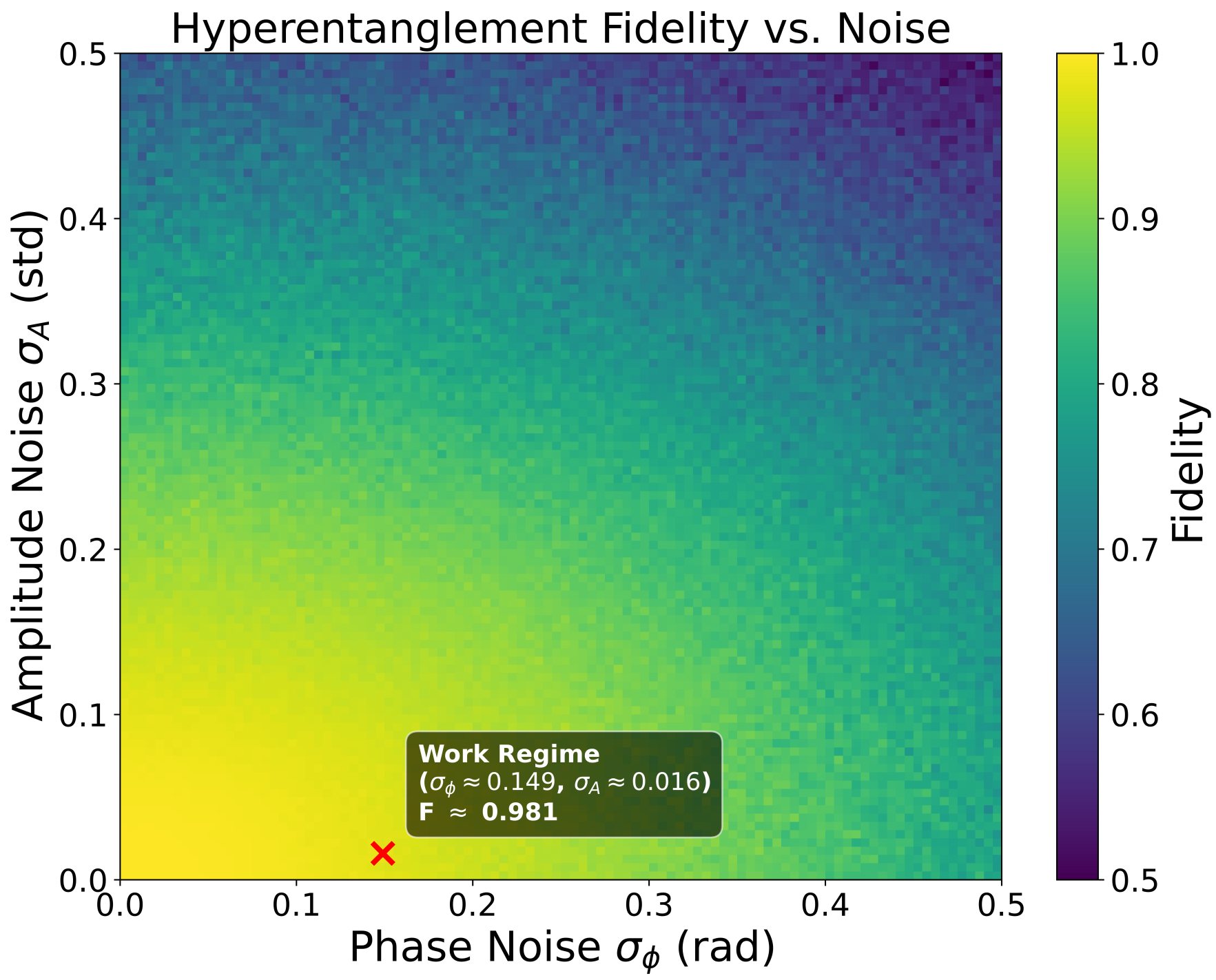

Figure S4: Hyperentanglement Fidelity Landscape - The color map indicates the fidelity of the generated state as a function of phase noise (σ_ϕ) and amplitude noise (σ_A). The red 'X' marks the estimated experimental work regime based on our noise analysis ($\sigma_\phi \approx 0.149$ rad, $\sigma_A \approx 0.016$).

The simulation confirms that the system is robust within the estimated experimental tolerances. At the calculated working point determined in the previous section, the fidelity remains high. As expected from the interference nature of the mode formation, the fidelity is more sensitive to phase errors (which disrupt the destructive/constructive interference patterns) than to amplitude errors (which primarily affect the relative weights of the superposition).

References

[1] G. Spektor, A. David, G. Bartal, M. Orenstein, and A. Hayat, "Spin-patterned plasmonics: towards optical access to topological-insulator surface states", Optics Express **23**, 32759 (2015).

[2] W. H. McMaster, "Polarization and the stokes parameters", American Journal of Physics **22**, 351–362 (1954).

[3] S. Tsesses, K. Cohen, E. Ostrovsky, B. Gjonaj, and G. Bartal, "Spin–orbit interaction of light in plasmonic lattices", Nano Letters **19**, 4010–4016 (2019).

[4] F. J. Garcia-Vidal, L. Martin-Moreno, T. W. Ebbesen, and L. Kuipers, "Light passing through subwavelength apertures", Reviews of Modern Physics **82**, 729–787 (2010).

[5] C. A. Volkert and A. M. Minor, "Focused ion beam microscopy and micromachining", MRS Bulletin **32**, Publisher: Springer, 389–399 (2011).
